# Preparation and Characterization of a Thin-Film Composite Membrane Modified by MXene Nano-Sheets

**DOI:** 10.3390/membranes12040368

**Published:** 2022-03-28

**Authors:** Yi Wang, Yuqi Nie, Chunhong Chen, Hongjie Zhao, Ye Zhao, Yujin Jia, Jun Li, Zhanguo Li

**Affiliations:** 1 State Key Lab of NBC Protection for Civilian, Beijing 102205, China; wangyi102205@sina.com (Y.W.); cch2022my@163.com (C.C.); zhaohongjieok@126.com (H.Z.); 2 Department of Military Installation, Army Logistics Academy of PLA, Chongqing 401331, China; nyq603394605@sina.com; 3 Chinese PLA General Hospital, Beijing 100853, China; yezhao2022@163.com (Y.Z.); yujin2022@163.com (Y.J.)

**Keywords:** TFC-FO membrane, MXene nano-sheets, internal concentration polarization

## Abstract

MXene nano-sheets were introduced into a thin-film composite membrane (TFC) to reduce the mass transfer resistance (concentration polarization) and improve the membrane performance. The process entailed dissolving the MXene nano-sheets in a membrane casting solution using the blending method and introducing them into the porous support layer to prepare a modified thin-film composite forward osmosis (TFC-FO) membrane. The results showed that the water contact angle decreased by about 16%, indicating that the hydrophilicity was strengthened, and the O/N ratio of the active selective layer decreased by 13%, indicating an increased degree of crosslinking, thereby demonstrating that the introduction of MXene nano-sheets changed the properties of the membrane and played a positive role in its physicochemical properties. In contrast to the unmodified TFC-FO membrane, the modified membrane had a slightly higher reverse solute flux, while its water flux increased by about 80%. Its specific reverse osmosis flux was also significantly optimized (only 0.63 g/L). In conclusion, adding MXene nanosheets to TFC-FO membranes led to the modified membranes with better mass transfer, lessened internal concentration polarization (ICP), and better osmotic separation.

## 1. Introduction

With the increasing impact of the global shortage of water resources on human society, the protection of freshwater resources is especially important. Membrane separation technology has become one of the main technical means of addressing freshwater issues and improving water recycling efficiency and has yielded remarkable results in the area of seawater desalination. Among the membrane types currently used, thin-film composite (TFC) membranes are among the most widely studied and frequently used [1] and are considered the most effective membranes for desalination. Generally, interfacial polymerization is carried out on the porous support layer formed by phase transformation to form an asymmetric membrane composed of the porous support layer and an active selective layer, which has the characteristics of high selectivity and low energy consumption.

The main membrane processes used in the production of TFC membranes are reverse osmosis (RO) and forward osmosis (FO). In the past, reverse osmosis technology was widely used all over the world and was considered the most effective desalination process. However, its high energy consumption was a technical bottleneck, so the focus shifted to FO membrane separation technology. Compared with the traditional pressure-driven membrane separation process, FO technology has demonstrated the outstanding advantages of a high cycle recovery rate, low system energy consumption possibility, less membrane fouling propensity, and low operating pressure input. Thus, it has excellent potential for future application [2].

Currently, FO technology is widely used and has achieved good results in green production, wastewater treatment, food processing, biomedicine, and other fields [3]. To further improve the filtration efficiency of FO technology, researchers have studied the preparation and application of TFC-FO membranes. However, concentration polarization has been a major limiting factor, which hinders the mass transfer process, greatly reducing the performance of the TFC-FO membrane and resulting in the actual water flux being far less than the theoretical water flux. This has greatly limited the development of FO technology [4]. Previous studies have shown that the introduction of nanomaterials into TFC membranes can effectively reduce mass transfer resistance and alleviate concentration polarization [5]. Therefore, researchers have begun to add nanomaterials to the porous support layer or active selective layer to improve the mass transfer process, alleviate concentration polarization, and improve membrane performance. For example, Xiao Tong blended carbon nanoparticles with polyethersulfone (PES) to prepare a conductive thin-film nanocomposite membrane with improved anti-fouling ability. The gradual addition of modified materials was found to significantly enhance the conductivity of the membrane. Under the conditions of additional voltage and no voltage, the water flux of the modified membrane remained close to its pristine value after 8 h, indicating improved anti-fouling ability [6]. Niksefat used silica nanoparticles as additives to improve the performance of TFC membranes, dispersing the nanoparticles into the aqueous phase during the interfacial polymerization reaction to prepare an active layer. The results showed that the implantation of SiO_2_ improved the interfacial polymerization rate of FO and enhanced its hydrophilicity. The modified FO membranes had high water flux and low reverse solute flux, achieving a maximum water flux of approximately 36 LMH [7]. Due to the interaction between the porous support layer and the active selective layer, there was an inseparable relationship. Some researchers have modified both layers at the same time. For example, Masood simultaneously added halloysite nanotubes (HNT) to the polysulfone support layer using the mixing method and added functional nano cellulose (NCC) by mixing it into the polyamide active selective layer to prepare modified TFC-FO membranes. The results showed that the addition of HNT increased the growth of the finger pore structure of the support layer and that NCC made the surface of the active selective layer more compact. With both additions, the permeability test results showed that the water flux was around 140% higher than that of the unmodified membranes, constituting a breakthrough in this direction [8]. This shows that choosing one or two nanomaterials and changing the membrane structure can improve the performance of the TFC-FO membrane to a certain point.

At present, numerous new nanomaterials are being used in membrane separation technology. Due to their chemical function and special physical structure, the properties of the modified membranes are significantly improved [9].Among these new nanomaterials, two-dimensional transition metal carbide MXene nano-sheets have been widely used in wastewater treatment, seawater desalination, and gas separation due to their thermal stability, excellent film-forming ability, and high hydrophilicity, constituting a breakthrough in modified membrane research [10,11]. For example, Huang et al. mixed magnetic nickel MXene with polyethersulfone (PES) to prepare a modified ultrafiltration membrane. The results showed that the modified PES membrane was 2.5 times higher than the unmodified membranes in the water flux, and the flux recovery of the humic acid solution reached 99.8% [12]. Alfahel prepared an MXene-modified nanofiltration membrane by mixing MXene with cellulose acetate via blending and crosslinking. The addition of MXene was found to improve the hydrophilicity and anti-fouling performance of the membrane. Its anti-fouling ability proved superior to that of commercial membranes [13]. Taking advantage of the scalability of MXene and the special structure of the TFC-FO membrane, Wu prepared a modified TFC-FO membrane by constructing an MXene nano interlayer between the porous support layer and the active selective layer and found that the water flux was improved due to the promotion of MXene layer spacing on moisture particles [14]. It can be seen that MXene has been applied to membrane application fields, but for the field of forward osmosis membrane, only the influence of constructing an MXene interlayer on the TFC-FO membrane has been studied. Few researchers have systematically studied how the addition of MXene to the porous support layer, the active selective layer, or both affects the performance of the whole membrane. Thus, further research is urgently needed to explore the application potential of MXene in the TFC-FO membrane.

Given the key influence of the structural characteristics of the porous support layer on the performance of the active selective layer and the whole TFC-FO membrane, and the effect of internal concentration polarization (ICP) on the permeation mass transfer process, which mainly takes place in the porous support layer, this paper describes the use of the blending modification method to modify the TFC-FO membrane porous support layer with different addition amounts of MXene nano-sheets. State-of-the-art characterization methods, including scanning electron microscopy (SEM), atomic force microscopy (AFM), contact angle (CA), Fourier transform infrared spectroscopy (FTIR), and X-ray photoelectron spectroscopy (XPS), were used to analyze and study the as-prepared modified TFC-FO membranes.

## 2. Materials and Methods

### 2.1. Experimental Materials

The raw materials used to prepare the porous support layer were polysulfone (PSf, average molecular weight of 22,000 Da) purchased from Sigma-Aldrich, St. Louis, MI, USA, polyvinylpyrrolidone (PVP, K30) and N-methyl pyrrolidone (NMP, analytical purity >99%) from Aladdin, Shanghai, China. The raw materials used to prepare the active selective layer were m-phenylenediamine (MPD, analytical purity >99.5%), n-Hexane (Chromatographic grade, >98%) purchased from Aladdin, Shanghai, China, and trimethyl chloride (TMC, >98%) purchased from Sigma-Aldrich, St. Louis, MI, USA. The modified material was MXene nano-sheets (single layer), which was provided by Suzhou Bei Ke 2D Materials Co., Ltd. Suzhou, China. In addition, the reagents for preparing the draw solution and feed solution were sodium chloride, which was purchased from Macklin, Shanghai, China, and self-made pure water (conductivity less than 5 µS/cm), respectively.

### 2.2. Preparation of the MXene Nano-Sheets Modified TFC-FO Membranes

#### 2.2.1. The Preparation of the Modified Porous Support Layer

**Figure 1:**
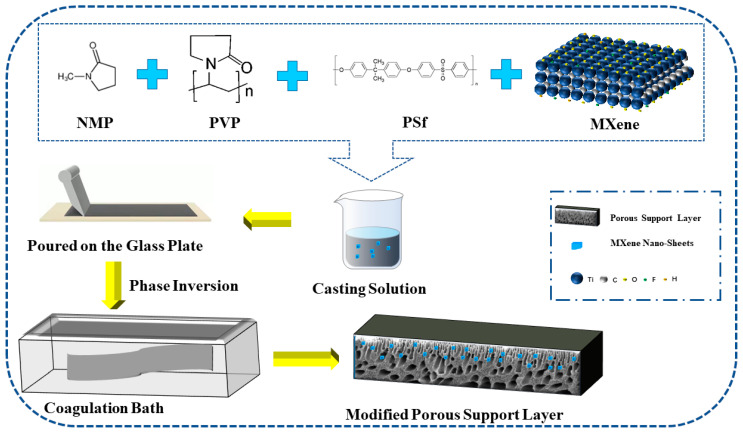
The porous support layer preparation by the phase inversion method.

The dried MXene nano-sheets were ultrasonically dispersed in pure water for more than 3 h to prepare the MXene dispersion solution. The PSf, PVP, and NMP were mixed to a ratio of 7:2:40 in a container and then fully stirred with a constant temperature magnetic stirrer for 24 h (25 °C) before being left to stand for 12 h to obtain a clear casting film solution. The casting solution was mixed with MXene dispersion solution in the container, and the mixture was fully stirred with a magnetic stirrer for 48 h. Ultrasonic dispersion was carried out alternately during the mixing process. The prepared mixture was evenly poured on a clean glass plate and then put into a coagulation bath for 30 min to prepare a modified porous support layer [15], as shown in Figure 1.

#### 2.2.2. Preparation of the Active Selective Layer

**Figure 2:**
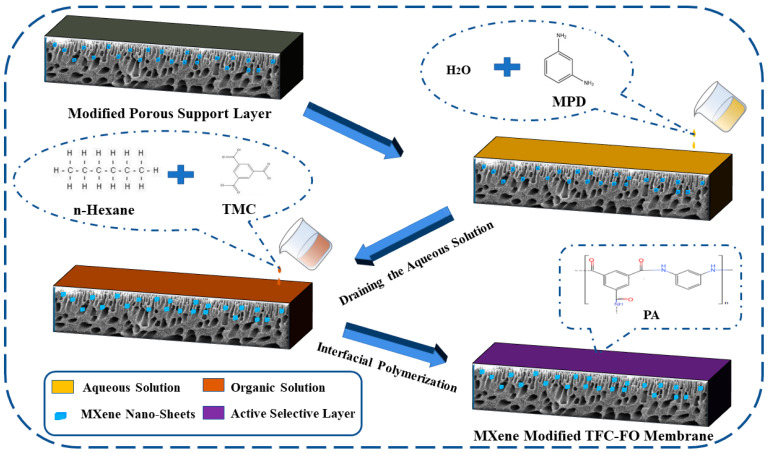
The active selective layer preparation by the interfacial polymerization reaction.

The active selective layer was prepared on the modified porous support layer by a classic interfacial polymerization reaction [16]. First, an MPD aqueous solution (the ratio of MPD to pure water was 2.0%) was poured on the porous support layer. After soaking for 2 min, the aqueous solution was drained and immersed in the TMC/n-Hexane solution (the ratio of TMC to n-Hexane was 0.13%) for 1 min. It was then placed in an oven for drying and finally rinsed with pure water to prepare the modified TFC-FO membranes. The addition amounts of MXene nano-sheets were 0%, 0.005%, 0.007%, 0.01%, and 0.03%, which correspond to the prepared TFC-FO membranes T-1, T-2, T-3, T-4, and T-5, respectively, as shown in Figure 2.

### 2.3. Characterization of the MXene Nano-Sheets

The surface morphology of MXene nano-sheets was observed by an emission scanning electron microscope (FEI inspect F50 FSEM, Hillsboro, OR, USA). The chemical element composition was measured by the ATR mode transforming the infrared spectrum of the spectrometer (Thermo Fisher Nicolet IS5, Chengdu, China) and X-ray electron spectroscopy (XPS, Thermo Scientific Escalab250Xi, Waltham, MA, USA).

### 2.4. The Characterization of the MXene Nano-Sheets Modified TFC-FO Membranes

Contact angle meter (CAM, Kruss DSA100, Hamburg, Germany), emission scanning electron microscope (FEI inspect F50 FSEM, Hillsboro, OR, USA), and atomic force microscope (dimension icon AFM, Billerica, MA, USA) were used to characterize the physical morphology of the modified TFC-FO membrane. The chemical element composition was measured by the ATR mode transforming the infrared spectrum of the spectrometer (Thermo Fisher Nicolet IS5, Chengdu, China) and X-ray electron spectroscopy (XPS, Thermo Scientific Escalab250Xi, Waltham, MA, USA).

### 2.5. Permeability Test of the MXene Nano-Sheets Modified TFC-FO Membranes

#### 2.5.1. Test Equipment

**Figure 3:**
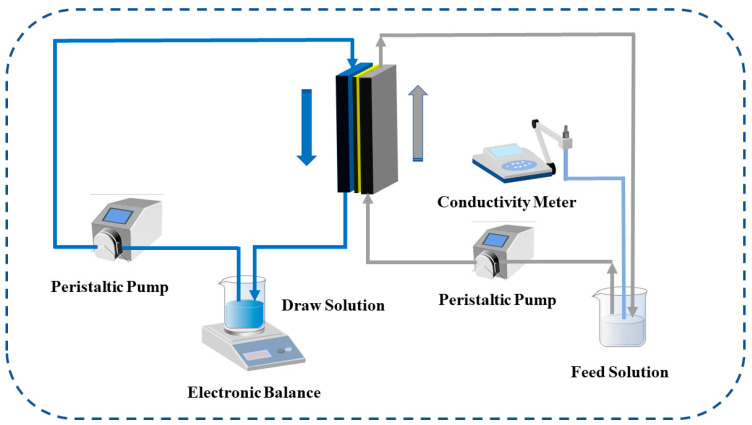
The schematic diagram of the TFC-FO membranes performance testing system.

The TFC-FO membrane test equipment consisted of a self-made membrane module, peristaltic pumps (Jie Heng, Chongqing, China), a conductivity meter (Lei Ci, Shanghai, China), and an electronic balance (APTECH Shenzhen, China). The effective test area of the membrane was 8 cm^2^, and 1M NaCl solution and pure water were used as the draw solution and feed solution, respectively. The change of the weight in the draw solution during the forward osmosis process was recorded by the electronic balance, and the change in the conductivity in the feed solution was recorded by the conductivity meter. The permeability test of the TFC-FO membrane included the AL-DS mode and the AL-FS mode, which were the active selective layer facing draw solution and the active selective layer facing feed solution, respectively. The cross-flow filtration method was adopted, as shown in the Figure 3.

#### 2.5.2. Test Parameters

Test indicators of the TFC-FO membranes included water flux (*J_w_*), reverse solute flux (*J_s_*), and specific reverse solute flux (*F_S_*) [17]. The water flux represents the permeability of the membrane, and a higher water flux means superior permeability. The reverse solute flux represents the interception performance of the membrane, and a low reverse solute flux means strong interception capacity. The specific reverse solute flux represents the comprehensive separation performance of the membrane, and the lower its value, the better the membrane selectivity.

The calculation formulas are:

(1) $$J_{w}\;=\frac{\Delta V}{A\cdot T}$$

where *J_w_* is the water flux of the TFC-FO membrane (unit: L·m^−2^·h^−1^), ΔV is the volume change of the draw solution (unit: L), *T* is the operation time of the forward osmosis process (unit: h), and *A* is the effective test area of the TFC-FO membrane module (unit: m^2^).

(2) $$J_{s}=\frac{C\times \left(Vc-Vs\right)}{A\cdot T}$$

where *J_s_* is the water flux of the TFC-FO membrane (unit: g·m^−2^·h^−1^), C is the solute concentration of the draw solution in the feed solution (unit: g/L), Vc is the initial feed solution volume (unit: L), Vs is the volume of permeated pure water (unit: L), *T* is the operation time of the forward osmosis process (unit: h), and *A* is the effective area of the TFC-FO membrane module (unit: m^2^).

(3) $$F_{S}\;=\frac{Js}{Jw}$$

where *F_S_* is the specific reverse solute flux of forward osmosis (unit: g·L^−1^).

## 3. Results and Discussion

### 3.1. Characterization of MXene Nano-Sheets

**Figure 4:**
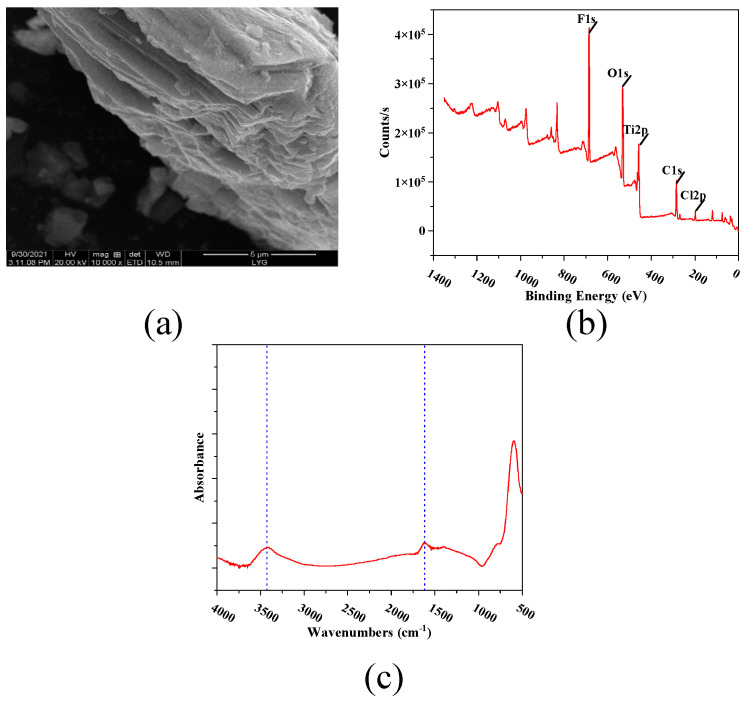
The characterization of MXene nano-sheets: (**a**) SEM micrograph, (**b**) XPS spectra, (**c**) Infrared spectrogram in the range of 400–4000 cm^−1^.

Figure 4a is the scanning electron microscope diagram of MXene nano-sheets. Through this diagram, it can be observed that it presented an overlapping structure, which was consistent with the test results of Shi et al. [18]. Figure 4b presents an XPS diagram of the MXene nano-sheets, which shows that the main elements in the MXene nano-sheets were C, O, Ti, and F. Figure 4c shows the Fourier infrared spectrum of the MXene nano-sheets. The figure shows characteristic peaks around 3400 cm^−1^ and 1650 cm^−1^, which were caused by the O-H and C=O vibrations in the MXene nano-sheets [19].

### 3.2. Characterization of the Physical Morphology of the TFC-FO Membranes Modified by MXene Nano-Sheets

**Figure 5:**
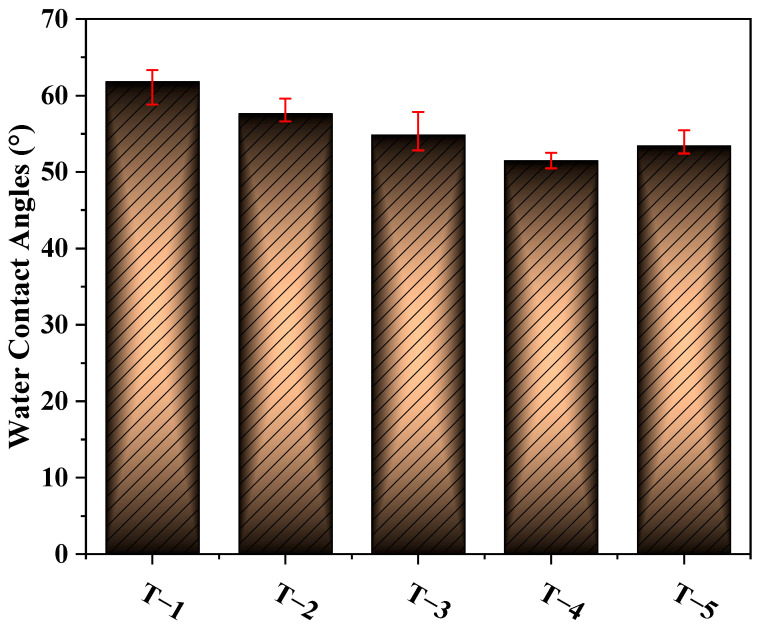
The water contact angles of the TFC-FO membranes with different amounts of MXene nano-sheets.

Figure 5 shows the effects of different MXene nano-sheet additions on the water CA of the TFC-FO membranes. It can be seen from the figure that the water CAs on the surface of membranes T-1 to T-5 were 61.7°, 57.6°, 54.8°, 51.4°, and 53.3°, respectively. The water contact angles on the surface of the TFC-FO membranes showed a downward trend. After the addition of MXene nano-sheets, the TFC-FO membranes had a more hydrophilic active selective layer. This was because the MXene nano-sheets contained a large number of O-H bonds of hydrophilic groups [20]. After being added to the casting solution by blending modification, the MXene nano-sheets were driven by the hydrogen bonds contained in water molecules and evenly distributed in the membrane during the phase transformation process. Therefore, the prepared porous support layer had a more hydrophilic membrane surface, which promoted the formation of a more hydrophilic active selective layer, strengthening the hydrophilicity of the prepared TFC-FO membranes. At the same time, the presence of a large number of C=O and N-H hydrophilic functional groups in the active selective layer further strengthened the hydrophilicity of the TFC-FO membranes. The subsequent contact angle showed a slight upward trend, which may be because the high concentration of MXene nano-sheets led to an increase in the viscosity of the casting solution, which was similar to the phenomenon of high polymer concentration [21], inhibiting the exchange rate between solvent and non-solvent in the non-solvent-induced phase separation process; this, in turn, affected the uniform distribution of MXene nano-sheets on the membrane surface, and finally led to a slight decrease in the hydrophilicity of the membrane. In addition, a more hydrophilic membrane matrix was also conducive to reducing the resistance in the mass transfer process and improving water flux.

**Figure 6:**
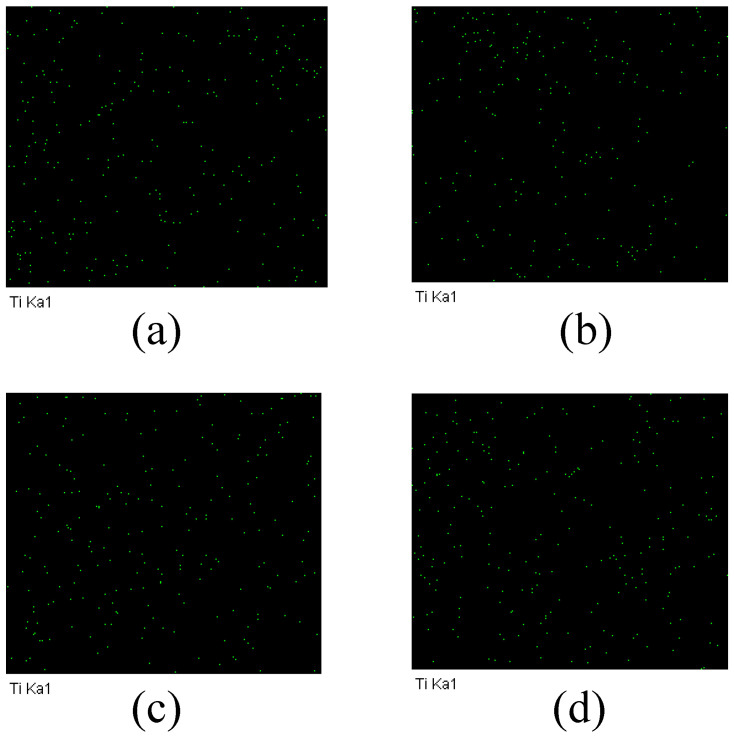
The EDX diagram of the TFC-FO membranes with different amounts of MXene nano-sheets: (**a**) T-2; (**b**) T-3; (**c**) T-4, and (**d**) T-5.

Since the main element in the MXene nano-sheets was Ti, the distribution of Ti can prove whether MXene nano-sheets were added to the porous support layer. Figure 6 shows the results of an EDX scan of Ti in the modified porous support layer. It can be seen that Ti was evenly distributed throughout the porous support layer, proving that MXene nano-sheets were successfully added to the porous support layer.

**Figure 7:**
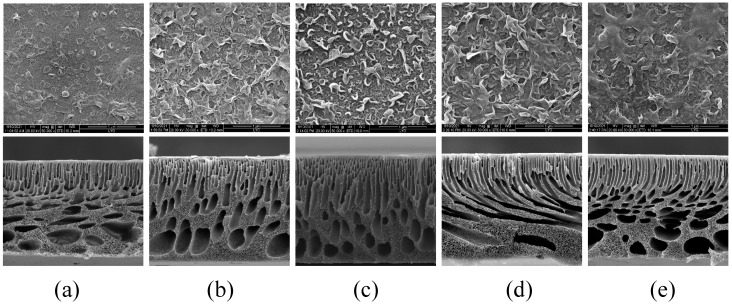
The SEM images of the TFC-FO membranes with different amounts of MXene nano-sheets: (**a**) T-1; (**b**) T-2; (**c**) T-3; (**d**) T-4, and (**e**) T-5.

Figure 7 shows SEM images of the surface and cross-section of the TFC-FO membranes prepared by adding different concentrations of MXene nano-sheets. As can be seen from the figure, not only did the addition of MXene nano-sheets directly impact the porous support layer, but it also indirectly affected the formation of the active selective layer. The plane SEM diagram showed that all membrane surfaces showed the typical morphology of the active selective layer and the structural characteristics of the “protuberance” [22]. Due to the limitations of the membrane structure and the surface hydrophilicity of the porous support layer, fewer aqueous phases participated in interfacial polymerization, so the morphological characteristics of the “protuberance” of the polyamide active selective layer were not obvious. Therefore, the “protuberances” in the active selective layer of the T-1 membrane were few and insignificant. When the MXene was added, the prepared TFC-FO membrane active selective layer immediately changed to present a more obvious “protuberance” structure, and the structural characteristics became more obvious. Due to the gradual increase in the MXene nano-sheet concentration, the sheets were evenly dispersed throughout the porous support layer, forming a more hydrophilic membrane surface with more aqueous phases, which intensified the interfacial polymerization reaction. However, when the MXene nano-sheet addition amount was increased to 0.03% due to the aggregation of excess MXene nano-sheets in the porous support layer, the aqueous phase attached to the porous support layer was also reduced accordingly, which indirectly affected the formation of the active layer. Therefore, the “protuberance” structure was significantly reduced.

The SEM cross-section images show that the as-prepared TFC-FO membranes were roughly composed of a thin, dense skin layer, a finger pore channel layer, and a sponge macropore structure at the bottom. Due to the good affinity between hydrophilic MXene nano-sheets and water, the rapid exchange of water and solvent may lead to the change of membrane cross-section morphology [21]. Compared with the TFC-FO membranes without MXene nano-sheets, all the TFC-FO membranes prepared with MXene nano-sheets showed varying degrees of membrane pore structure changes, which mainly resulted in the generation of more and longer finger pore channel structures and a reduction in sponge-like macropore structures. As the amount of added MXene nano-sheets increased, the finger pore structure grew longer. The finger pore structure of the porous support layer was longest when the addition of MXene nano-sheets reached 0.01%, indicating that this addition could promote the growth of finger pore structure in the resultant membrane. This was attributed to a large number of hydrophilic groups inside the MXene nano-sheets themselves, which lowered the thermodynamic stability of the membrane casting dope during the non-solvent-induced phase separation process, increasing the mass transfer rate between solvent and non-solvent, encouraging the formation of finger pore structures that provide water molecular channels, improving the mass transfer process, alleviating the ICP issue, and promoting the transport of water molecules [23]. The combination of these factors meant that the modified membranes performed better in terms of water flux. The specific impact on the water flux was further analyzed via the osmotic separation performance test. However, as more MXene nano-sheets were added, the pore structure of the T-5 membrane was significantly reduced. This may be similar to the agglomeration phenomenon caused by the addition of excessive MXene nano-sheets [24], which improved the viscosity of the casting solution and dynamically reduced the exchange rate between solvent and non-solvent in the non-solvent-induced phase separation process, thus hindering the formation of finger pore structures.

**Figure 8:**
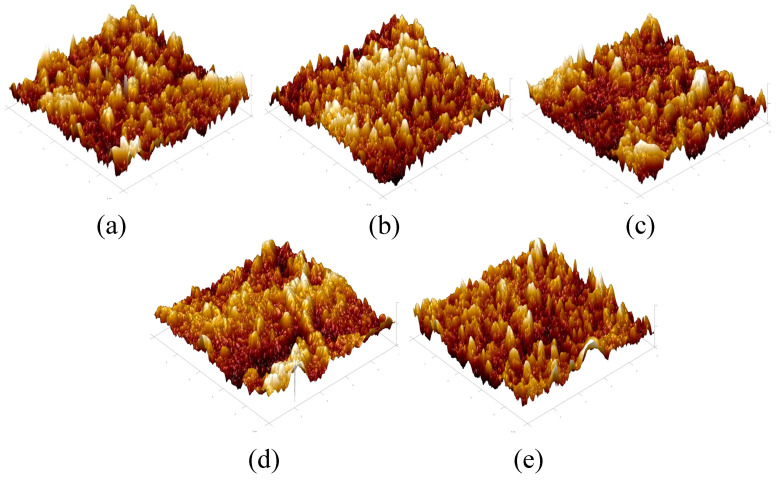
The AFM images of the TFC-FO membranes with different amounts of MXene nano-sheets: (**a**) T-1; (**b**) T-2; (**c**) T-3; (**d**) T-4, and (**e**) T-5.

The AFM images of the active selective layer of the TFC-FO membranes prepared by interfacial polymerization are shown in Figure 8. The morphology in the figure shows that all the membrane surfaces had a typical polyamide “valley ridge” structure, which also indicated that the TFC-FO membranes were successfully prepared. In addition, it can be seen that with the gradual addition of more MXene nano-sheets, the protrusion morphology in the figure became sharper, which meant that the contact area between the membrane surface and the water molecules increased.

Table 1 shows the surface roughness of the TFC-FO membranes modified by different MXene nano-sheets. It can be seen from the figure that the surface roughness of the TFC-FO membranes prepared on the porous support layer with different MXene nano-sheet additions was higher than that of the unmodified TFC-FO membranes and showed a gradual upward trend. One possible reason was that it increased the pore size and hydrophilicity of the porous support layer and promoted the reaction rate of aqueous phase diffusion to organic phase when a certain number of MXene nano-sheets were added [25], and the aqueous phase attached to the porous support layer’s membrane surface increased its participation in the interfacial polymerization reaction with the organic phase. The reaction was more intense, so the roughness of the polyamide active selective layer increased. However, the roughness later decreased due to the distribution of MXene nano-sheets in the porous support layer when the concentration of MXene nano-sheets continued to increase. Excessive particles led to agglomeration, destroying the membrane structure, and causing the roughness of the polyamide active selective layer to decrease synchronously.

### 3.3. Chemical Element Composition Characterization of the TFC-FO Membranes Modified by MXene Nano-Sheets

**Figure 9:**
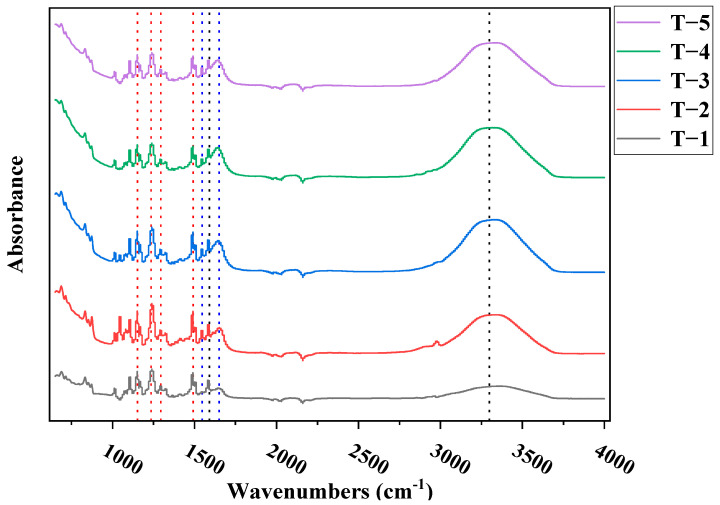
The ATR-FTIR spectra of the TFC-FO membrane with different amounts of MXene nano-sheets.

Figure 9 shows the test results of the FTIR spectra of membranes T-1 to T-5. It can be seen from the figure that all symmetrical O=S=O peaks (1148 cm^−1^), symmetrical C-O-C peaks (1237 cm^−1^), asymmetric O=S=O peaks (1294 cm^−1^), and CH_3_-C-CH_3_ peaks (1503 cm^−1^) of polysulfone membranes appeared. Moreover, a series of characteristic peaks and obvious characteristic peaks were observed at about 1610 cm^−1^, 1650 cm^−1^, and 1544 cm^−1^, which were respectively attributed to the aromatic ring, amide I band (C=O), and amide II band (C-H) in the active selective layer, indicating the success of the interfacial polymerization [26]. At the same time, in the range of 2000–4000 cm^−1^, with the addition of MXene nano-sheets, the characteristic peak of approximately 3300~3400 cm^−1^ gradually increased, which may be due to the hydrophilic groups contained in the MXene nano-sheets, resulting in a more hydrophilic porous support layer.

**Figure 10:**
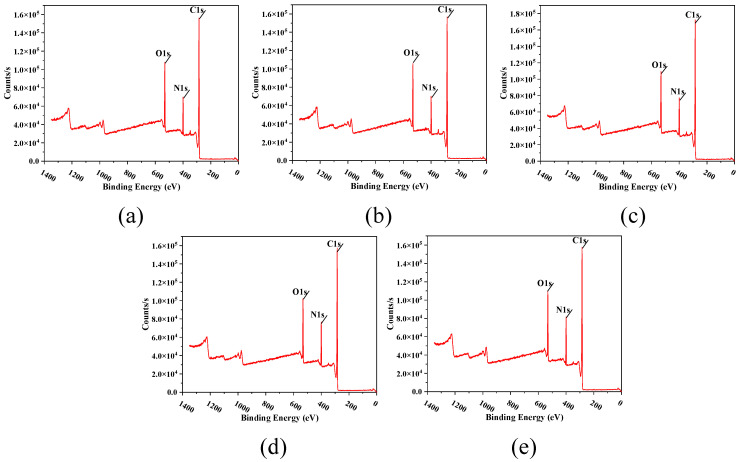
The XPS spectra of the surface of the TFC-FO membranes with different amounts of MXene nano-sheets: (**a**) T-1; (**b**) T-2; (**c**) T-3; (**d**) T-4, and (**e**) T-5.

Figure 10 shows the XPS test results of the prepared TFC-FO membranes modified with MXene nano-sheets. It can be seen that membranes T-1 to T-5 were mainly composed of oxygen, nitrogen, and carbon. Using Table 2, we can calculate the percentage content of C, O, and N. The crosslinking degree of the TFC-FO membrane surface was obtained by calculating the O/N ratio. The lower the O/N value, the higher the crosslinking degree of the membrane surface, and the higher the salt rejection rate of the active selective layer. Conversely, the higher the O/N value, the lower the crosslinking degree of the membrane surface, and the more unfavorable it was for the active selective layer to intercept salt ions [27]. The XPS results showed that the oxygen–nitrogen ratio of the membrane surface active selective layer first decreased and then increased with the addition of the MXene nano-sheets, and the crosslinking degree of the TFC-FO membranes modified by the MXene nano-sheets was higher than that of the unmodified TFC-FO membranes. The reason for the initial decrease and subsequent increase in the oxygen–nitrogen ratios could be that the initial addition of MXene nano-sheets (0.005%) had an immediate significant impact on the formation of the porous support layer, changed the membrane structure and the surface of the porous support layer, and indirectly played a positive role in the formation of the polyamide active selective layer generated by interfacial polymerization in the later stage, so the O/N ratio decreased greatly. When MXene nano-sheets were added continuously (0.007–0.01%), because the MXene nano-sheets were evenly distributed in the porous support layer, the membrane structure of the porous support layer was directly changed, enhancing the adhesion ability and diffusion rate of the aqueous phase on the surface of the porous support layer. This greatly promoted the interfacial polymerization reaction, and the O/N ratio of the generated polyamide active selective layer continued to decrease significantly, leading to a substantial increase in the degree of crosslinking. However, when the addition amount of MXene nano-sheets reached 0.03%, a large number of excessive MXene nano-sheets gathered in the porous support layer, destroying the membrane structure of the porous support layer. Simultaneously, due to the synchronous reduction in hydrophilicity, the aqueous phase attached to the surface of the porous support layer decreased, and the corresponding degree of reaction with the organic phase decreased, which hindered interfacial polymerization and led to an increase in the O/N ratio and a decrease in the degree of crosslinking of the polyamide active selective layer.

### 3.4. Permeability Test of the MXene Nano-Sheets Modified TFC-FO Membranes

It can be seen that the TFC-FO membranes modified with MXene nano-sheets had obvious changes in terms of surface physical morphology and chemical element composition. TFC-FO membranes modified with different contents were tested to see how well they could pass through water. This was to see how these changes would affect the performance of the membrane.

#### 3.4.1. Effects of MXene Nano-Sheets on the Water Flux of the TFC-FO Membranes

**Figure 11:**
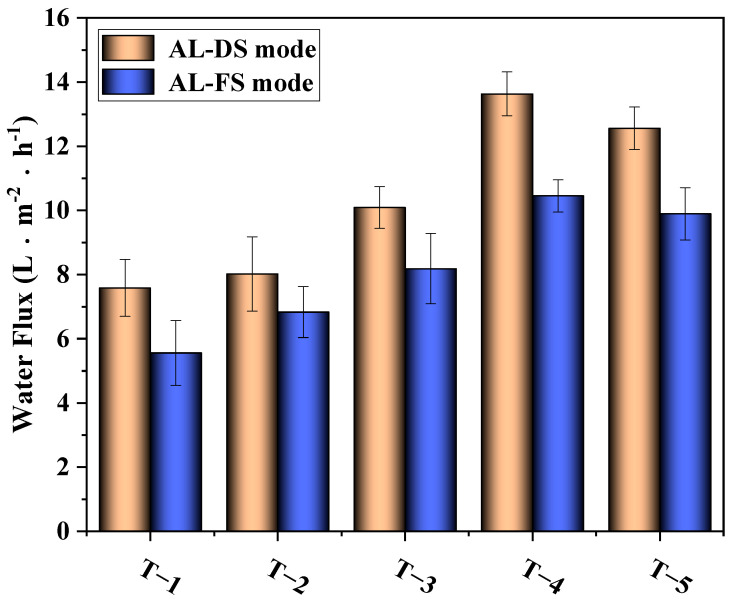
The effects of different amounts of MXene nano-sheets on the water flux of the TFC-FO membranes.

The results of the water flux test are depicted in Figure 11. The figure shows that with the increase in MXene nano-sheet content, the water flux of the TFC-FO membranes first increased and then decreased. When the addition amount of MXene nano-sheets was 0.01%, the water flux was the highest, reaching 13.63 L·m^−2^·h^−1^ (AL-DS mode) and 10.45 L·m^−2^·h^−1^ (AL-FS mode). According to the above analysis of physical structure and chemical composition, when the addition amount of MXene nano-sheets was within a certain range (0.005–0.01%), the MXene nano-sheets were evenly distributed and dissolved in the casting solution. On the one hand, the addition of MXene nano-sheets reduced the membrane contact angles and generated more finger pore structures. The reduction in the contact angles meant the enhancement of membrane hydrophilicity, which improved the mass transfer resistance and enhanced the affinity of TFC-FO membranes for water molecules, and the finger pore structure had a lower S value than the sponge macropore structure of the original membrane [28]. The lower the S value, the more conducive it was to alleviating the ICP and improving the mass transfer process, and the finger pore structure was shorter in the path than the sponge structure, which was theoretically conducive to reducing the ICP [29]. Based on the above two points, the addition of MXene nano-sheets reduced the mass transfer resistance of the membrane structure and was conducive to the transfer of water molecules in the membrane. On the other hand, due to the structural characteristics of MXene nano-sheets, they were evenly distributed in the porous support layer, providing additional transmission channels for the passage of water molecules and improving the water flux to a certain extent [30,31]. However, when the addition amount of MXene nano-sheets reached 0.03%, the high concentration caused agglomeration to occur in the membrane. The viscosity of the casting solution increased, forming a membrane pore structure that was not conducive to the passage of water molecules or directly blocked the finger pore structure. At the same time, the decrease in hydrophilicity also led to a decrease in the affinity of the TFC-FO membranes for water molecules in solution. Finally, the water flux decreased synchronously.

#### 3.4.2. Effects of MXene Nano-Sheets on the Reverse Solute Flux of the TFC-FO Membranes

**Figure 12:**
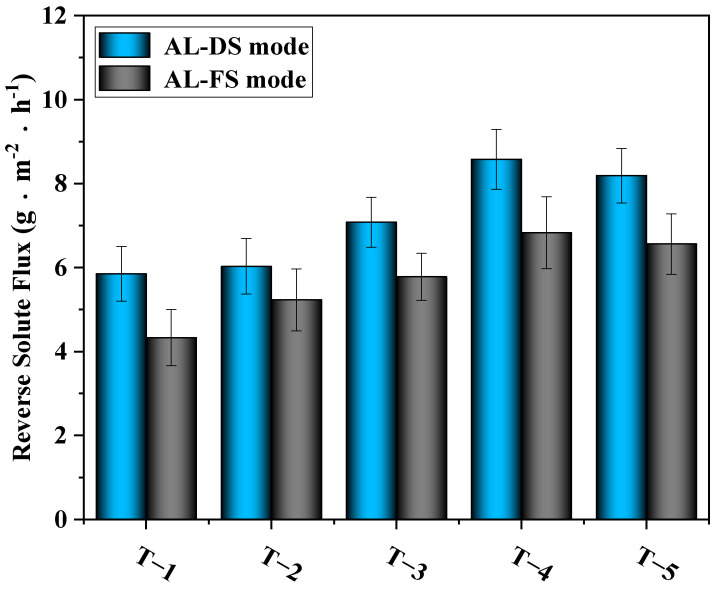
The effects of different amounts of MXene nano-sheets on the reverse solute flux of the TFC-FO membranes.

Figure 12 shows the results of the test of reverse solute flux. It can be seen from the figure that with the addition of MXene nano-sheets, the reverse solute flux of the modified TFC-FO membranes first increased and then decreased slightly. The highest reverse solute flux occurred when the addition amount of MXene nano-sheets was 0.01%, reaching 8.58 g·m^−2^·h^−1^ (AL-DS mode) and 6.83 g·m^−2^·h^−1^ (AL-FS mode). According to the above physical and chemical characterization, when the addition amount of MXene material was within a certain range (0.005–0.01%), although the addition of MXene material promoted the formation of an active selective layer, improved the crosslinking degree of the polyamide layer, and was conducive to the interception of salt, the MXene nano-sheets themselves produced swelling effects [32,33]. Under the influence of restrictive factors, the MXene nano-sheets had a unique adsorption effect on monovalent cations [30]. This occurred when the Na^+^ ions in the draw solution passed through the nanochannel formed by the MXene nano-sheets. Due to the selection of size and charge, Na^+^ ions were quickly adsorbed and intercalated into the MXene nano-sheets layer. With the electrostatic interaction between Na^+^ ions and negatively charged MXene nano-sheets, more Na^+^ ions were adsorbed, thus expanding the MXene layer. Not only did this create conditions for the rapid passage of water molecules, but it also facilitated the passage of a large number of salt ions. However, when the addition amount of MXene nano-sheets reached 0.03%, the reverse solute flux decreased, because, on the one hand, the excessive addition of MXene nano-sheets broke the finger pore structure in the porous support layer. On the other hand, as the concentration of MXene nano-sheets rose, the agglomeration phenomenon in the pore support layer blocked the formed pore channels and destroyed the extra nano channels that were formed since the swelling effect of MXene nano-sheets. This caused the final reverse solute flux to go down.

#### 3.4.3. Effects of MXene Nano-Sheets on the Specific Reverse Solute Flux of TFC-FO Membranes

**Figure 13:**
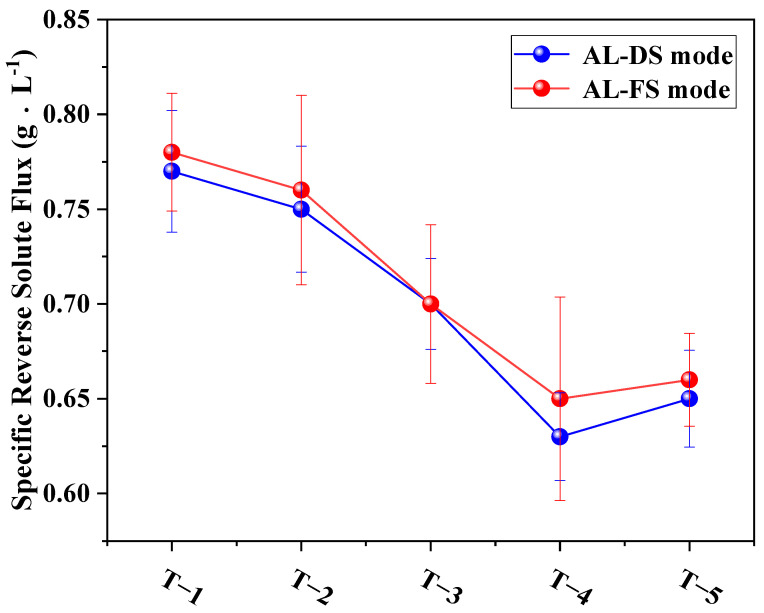
The effects of different amounts of MXene nano-sheets on the specific reverse solute flux of the TFC-FO membranes.

Figure 13 shows the ratio of water flux and reverse solute flux under two different modes (AL-DS and AL-FS), which reflect the separation performance of the TFC-FO membranes. As shown in the figure, the specific reverse solute flux of the modified membrane under both the AL-DS and AL-FS modes showed a consistent downward trend compared with the specific reverse solute flux value of 0.77 g·L^−1^ (AL-DS mode) and 0.78 g·L^−1^ (AL-FS mode) of the unmodified membrane, thus a better optimization was achieved. A comparison of the results showed that an addition amount of 0.01% resulted in the best membrane separation performance.

## 4. Conclusions

With the aim of achieving improved water flux in the process of TFC membrane filtration, this paper assesses the effects of MXene nano-sheets on TFC-FO membranes using the FO process. Tests of SEM, AFM, CA, FTIR, XPS, and osmotic separation performance were applied to systematically analyze the modified TFC-FO membranes. The successful introduction of MXene nano-sheets and their positive effects on membrane performance were demonstrated. The results showed that the surface morphology, roughness, hydrophilicity, and degree of crosslinking of the modified TFC-FO membranes changed in accordance with the variation in the introduction concentration of MXene nano-sheets. Compared with the unmodified TFC-FO membranes, the modified TFC-FO membranes had higher hydrophilicity, higher roughness, and a higher degree of crosslinking. Because of the more hydrophilic structure and the formation of more finger pore channels, the FO mass transfer process was improved, and the ICP effect was alleviated, which greatly improved the water flux. In the AL-DS mode, when the addition amount was 0.01%, the water flux of the FO membrane was 13.63 L·m^−2^·h^−1^, the reverse solute flux was 8.58 g·m^−2^·h^−1^, and the membrane separation performance was relatively good, indicating good application potential in the field of membrane separation. However, due to the swelling effect of the MXene nano-sheets and the agglomeration factor, further improvement of the performance of the TFC-FO membranes was limited, highlighting an important avenue for further research on process optimization.

## Figures and Tables

**Table 1:** The surface roughness of the TFC-FO membranes with different amounts of MXene nano-sheets.

TFC-FO Membranes	Ra (nm)	Rms (nm)
T-1	23.9	34.6
T-2	32.4	40.3
T-3	35.7	45.3
T-4	40.2	53.4
T-5	38.3	51.2

**Table 2:** The elements composition and O/N ratios of the TFC-FO membranes with different amounts of MXene nano-sheets.

TFC-FO Membranes	C Content (%)	O Content (%)	N Content (%)	O/N Value
T-1	75.41	13.91	10.68	1.30
T-2	74.82	13.68	11.39	1.20
T-3	73.93	13.84	12.08	1.15
T-4	74.43	13.48	11.97	1.13
T-5	72.21	14.84	12.78	1.16
